# Evaluation of the retinal and choroidal microvasculature changes in cases of sarcoid and tuberculosis-associated posterior uveitis using OCT angiography

**DOI:** 10.1007/s10792-022-02464-6

**Published:** 2022-08-27

**Authors:** Lameece Moustafa Hassan, Ashgan Asaad, Zeinab ElSanabary, Maha M. Youssef

**Affiliations:** 1 Cairo University, 3 Road 217, Degla Maadi, Cairo, 11431 Egypt; 2 Ministry of Health, Cairo, Egypt

**Keywords:** OCTA, FFA, Ocular sarcoidosis, Intraocular tuberculosis, Uveitis, Capillary vessel density

## Abstract

**Purpose:**

Using optical coherence tomography angiography (OCTA) to evaluate retinal microvascular changes in sarcoid and tuberculous (TB) posterior uveitis.

**Methods:**

Cross-sectional observational study includes 30 eyes. FFA and OCTA images were acquired. OCTA images were analyzed for areas of capillary hypo-perfusion, disorganization of the superficial and deep capillary plexuses (SCP and DCP) and intraretinal cystoid spaces and for measuring the size of the foveal avascular zone and vessel density (VD) in the SCP and DCP.

**Results:**

A total of 11 eyes were associated with TB and 19 with sarcoidosis. By OCTA, 100% had areas of capillary non-perfusion, 36.7% choroidal voids, 30% disorganization of the SCP and DCP and 26.6% intraretinal cystoid spaces. The VD of the DCP was significantly lower in the TB group. On comparing OCTA and FFA, parafoveal ischemia was detected more frequently on OCTA and macular edema more frequently on FFA (*P* =  < 0.001). The BCVA was not significantly correlated with the VD of the SCP or DCP.

**Conclusion:**

OCTA can be used in detection of early microvascular changes, segmenting retinal layers and localizing abnormalities. The presence of these changes may aid in the diagnosis of TB and sarcoid uveitis, for prognosis, follow-up and may be the only choice when FFA is contraindicated.

## Introduction

Sarcoid and tuberculous uveitis are common causes of chronic granulomatous inflammation, which can manifest as both anterior and posterior uveitis and result in significant visual disability from their chronic inflammatory sequelae [1]. Granulomatous uveitis most commonly results from sarcoidosis, a multifactorial syndrome stemming mainly from immune dysregulation [2], or a systemic infection, commonly tuberculosis (TB), which may affect the eye only (isolated ocular TB) or is part of a systemic condition [3].

Over many decades, the gold standard modality for the imaging of the retinal and choroidal vasculature and their pathologies, especially in uveitis, has been fundus fluorescein angiography (FFA). However, it is an invasive procedure, requiring the injection of intravenous dyes, which may be poorly tolerated and associated with rare serious side effects. It is also time-and effort-consuming, and leakage with intravenous (IV) FFA can obscure morphological vascular details and window defects can prevent accurate analysis of retinal details. Moreover, this modality provides two-dimensional evaluation of the retina and choroid and from them we are unable to identify the level of vascular abnormalities. Thus, it is impractical to repeatedly use angiography for patients to evaluate disease activity and progression [4]

Optical coherence tomography angiography (OCTA), employs amplitude or phase decorrelation to detect blood flow without the need for intravenous dye administration [5]. OCTA allows the study of micro vascular changes, and thus may provide an important tool in the evaluation of inflammatory eye diseases, as the vascular changes in the iris, choroid, and retina play an important role in the pathophysiology of ocular inflammatory diseases [4]. Moreover, OCTA image acquisition is easy, fast and non-invasive, limiting the risk of side effects for the patient. Depth-resolving capability gives important evaluation of the deep retinal capillary plexus, which may be targeted in retinal vascular or inflammatory diseases [6, 7].

The aim of this study is to evaluate the retinal and choroidal microvasculature qualitative and quantitative changes in eyes with sarcoid and tuberculous posterior uveitis using optical coherence tomography angiography (OCTA) and compare these changes with their corresponding fundus fluorescein angiography (FFA) findings and to correlate these changes with the disease type, treatment and activity.

## Methods

This is a cross-sectional observational study including 30 eyes of 30 patients (19 sarcoid and 11 tuberculous patients with posterior uveitis). Patients were recruited from the uveitis subspecialty clinic at Kasr ElAiny Hospital, Cairo University. The study was conducted during the period between December 2019 and July 2020.

## Statement of ethics

Cairo University ethical committee approval was obtained (N-24-2019) and the study followed the tenets of the Declaration of Helsinki. A written consent was taken from all patients participating in the study.

This study included patients with posterior uveitis or panuveitis secondary to sarcoidosis or tuberculosis. We excluded patients with other coexisting retinal diseases, eyes with refractive error of 6 diopters or more and eyes with dense media opacities obscuring imaging or lowering image resolution.

Detailed medical history was taken, and complete ophthalmological examination was conducted including visual acuity (best corrected visual acuity-BCVA, recorded in decimal notation), intraocular pressure measurement and slit lamp examination of the anterior segment, and dilated fundus examination was carried out using a binocular indirect ophthalmoscope and slit-lamp biomicroscopy, as is the routine for any uveitis patient on diagnosis and in follow-up.

The diagnosis of posterior uveitis or pan uveitis in all patients was made according to the SUN (Standardization of Uveitis Nomenclature) classification, grading of activity was done according to SUN classification as well [8]. Eyes with vitreous haze grading of >  + 0.5, with or without active choroidal or retinal inflammatory lesions, were considered active and this activity was confirmed by FFA findings. Ocular sarcoidosis was diagnosed according to the international workshop on ocular sarcoidosis (IWOS) criteria [9] and intraocular tuberculosis was diagnosed according to the criteria of classification of intraocular tuberculosis [10]. The consensus on the etiology, anatomical location and state of activity was made by two different uveitis consultants (MY and LH). Laboratory and radiological work up was done guided by the clinical condition of the patients.

Patients were classified according to specific systemic treatment into treated or non-treated (treatment naïve) patients. Treated sarcoid patients were those who received or continue to be under systemic steroid therapy with or without other immunosuppressive drugs and treated tuberculous patients included those who had completed anti-tuberculous treatment (ATT) for at least 6 months with or without steroid therapy.

Fundus photography and FFA were done for all patients using the TOPCON (TRC-50DX, Topcon Medical System Inc., 2015). FFA images were analyzed for vasculitis, focal or multifocal choroiditis, macular edema, optic disc leakage and areas of ischemia (peripheral capillary dropout or macular ischemia).

OCT angiography images were acquired of a 6 × 6 mm area in the central macula from all patients using RTVue XR Avanti (AngioVue, Optovue Inc, Fremont, California, USA). The flow imaging was based on Split-Spectrum Amplitude Decorrelation Angiography (SSADA).

Detection of the following qualitative parameters was done: areas of capillary non-perfusion/hypo perfusion, capillary changes (capillary dilatation, telangiectasia, shunting vessels and areas of rarefied capillaries), disorganization of the superficial and deep capillary network (localized or diffuse loss of the normal architecture of the capillary network), intraretinal cystoid spaces (defined as round black areas without any decorrelation signal and confirmed by both structural OCT and corresponding en face images) and choroidal affection in the form of choroidal voids.

OCTA was used to measure the size of the foveal avascular zone (FAZ) (mm^2^) and capillary vessel density (VD) in both the superficial and deep capillary plexuses at 9 areas grid-based vessel density (%). The foveal region was defined as the central 1 mm, parafoveal 1–3 mm and perifoveal region 3–6 mm according to the ETDRS Grid. Three captures were taken in the same setting and the mean VD was used.

All images were analyzed by the same consultant (ZS) to avoid interobserver variations. OCTA images were compared with both sequential en face images and SD OCT images to detect areas of signal loss and to differentiate vitreous opacities and artifacts with back shadowing from areas of non-perfusion.

Whole image capillary VD < 50% was considered as ischemia, according to the OCTA normative data for vascular density in the superficial and deep capillary plexuses of healthy adults determined by Coscas et al. [11]*.*

## Statistical methods

Microsoft excel 2013 was used for data entry and the statistical package for social science (SPSS version 24) was used for data analysis. Simple descriptive statistics (arithmetic mean, median and standard deviation) were used for summary of normal quantitative data. Bivariate relationship was displayed in cross-tabulations and comparison of proportions was performed using the Chi-square and Fisher’s exact tests where appropriate T-independent was used to compare normally distributed quantitative data and Mann–Whitney for skewed data. *P* value less than 0.05 was considered statistically significant.

## Results

This study included 30 eyes of 30 patients. In patients with bilateral affection, the eye with clearer media, allowing higher quality of imaging, was chosen.11 out of the 30 eyes were diagnosed as probable TB uveitis (36.7%) and 19 (63.3%) eyes were diagnosed as presumed sarcoid uveitis.

The mean BCVA (in decimal notation) of the TB group patients was 0.36 ± 0.24 SD, while it was 0.4 ± 0.29 SD in the sarcoidosis group, without any statistical difference.

By clinical examination and confirmed by fluorescein angiography, activity was detected in 5 (45.5%) of the eyes with TB uveitis and 6 eyes (31.6%) with sarcoid uveitis (with a statistically insignificant difference) (Tables 1, 2). Phenotypes of both the sarcoid and TB cases were divided between posterior uveitis in the form of multifocal choroiditis and/or retinal vasculitis or panuveitis with associated multifocal choroiditis and/or vasculitis. Three cases of retinal vein occlusion were noted; however no cases of serpiginous choroiditis or large solitary granulomas were encountered. As for the sarcoid associated cases, one case had an associated large nasal peripapillary granuloma (not encroaching on the macula).

**Table 1:** Demographic data and clinical findings in patients with TB associated uveities

	Count (*n* = 11)	%
***Gender***		
Male	3	27.3
Female	8	72.7
***Treatment***		
Treated	7	63.6
Untreated	4	36.4
***Activity***		
Inactive	6	54.5
Active	5	45.45
***Clinical patterns***		
Vasculitis	8	63.6
Multifocal choroiditis	4	36.4
Optic disc edema	2	18.2

**Table 2:** Demographic data and clinical findings in patients with sarcoidosis associated Uveitis

	Count (*n* = 19)	%
***Gender***		
Male	0	0
Female	19	100
***Treatment***		
Treated	15	78.9
Untreated	4	21
***Activity***		
Inactive	6	31.6
Active	13	68.4
***Clinical patterns***		
Vasculitis	6	31.6
Multifocal choroiditis	5	26.3
Optic Disc edema	7	36.8

### OCTA findings in all patients

**Fig. 1:**
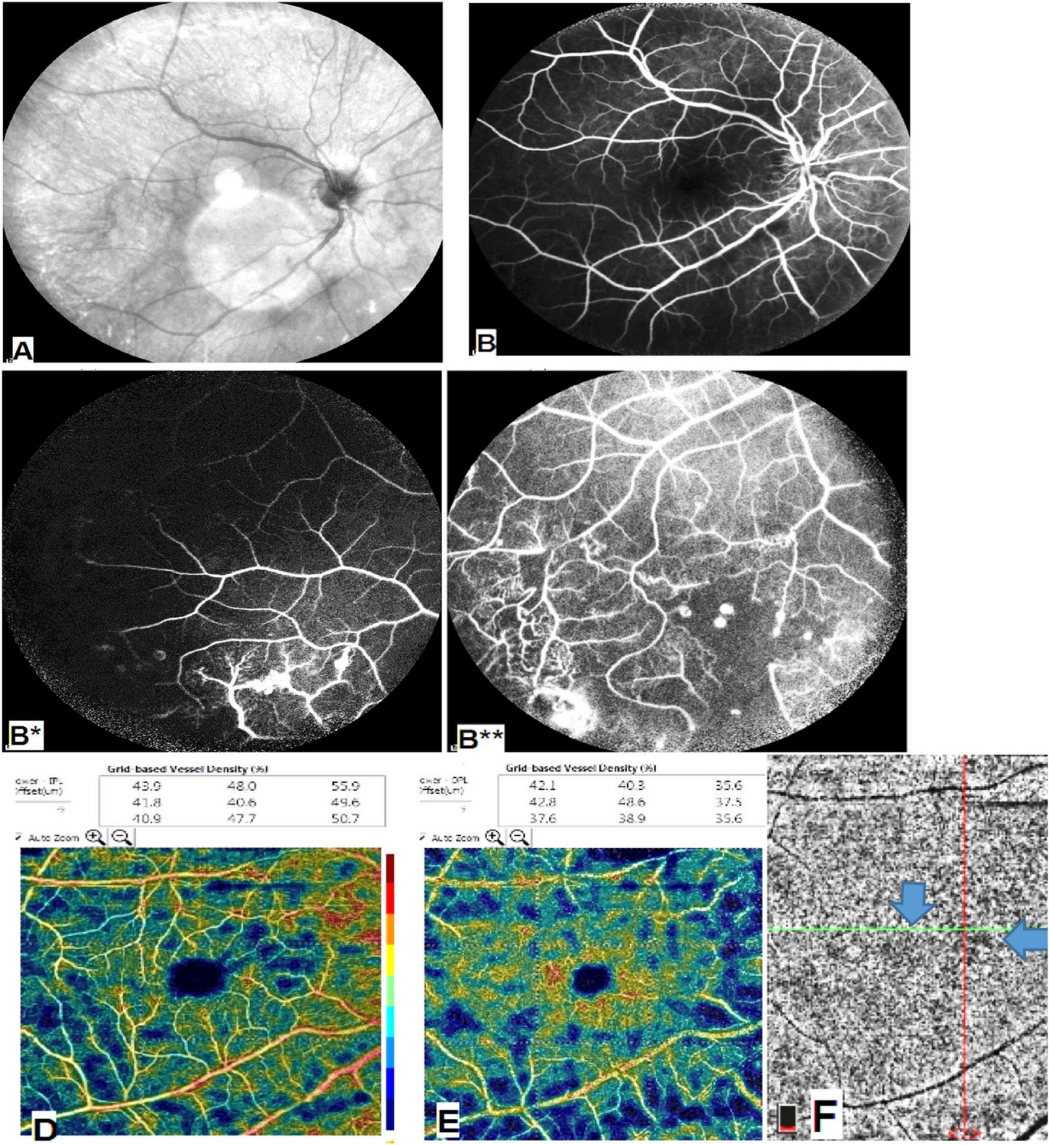
Patient with active tuberculosis. **A** Infra-red shows peripheral attenuated vessels. **B** FFA shows peripheral vasculitis, telangiectasia, peripheral choroidal lesions and peripheral ischemia. **C** Corresponding structural B-scan is normal. **D**, **E** SCP and DCP respectively show ischemia more in the deep capillary plexus. **F** Choriocapillaris layer shows areas of choroidal voids (arrows).

**Fig. 2:**
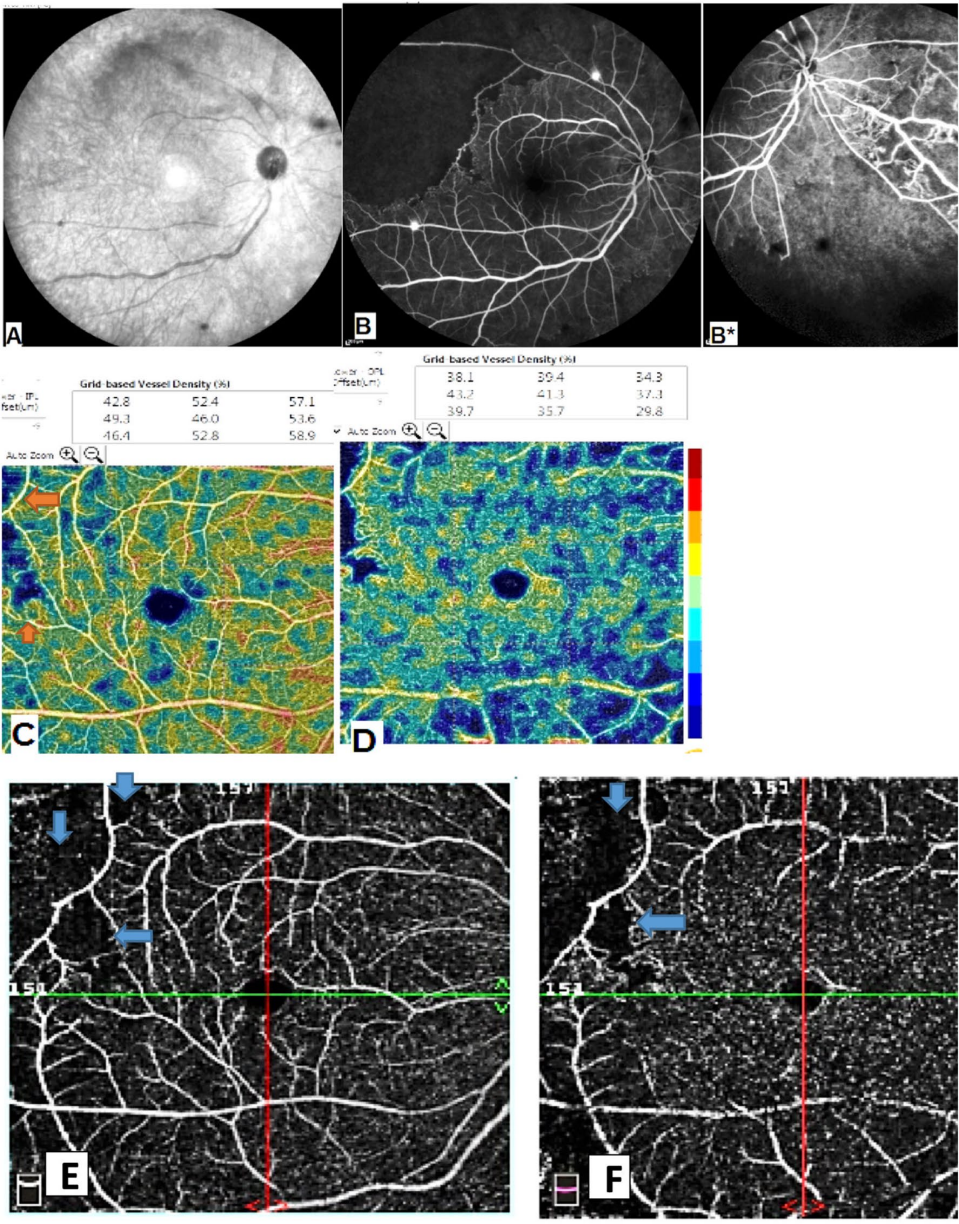
Patient with active tuberculosis. **A** Infra-red photo shows upper temporal ischemia and retinal hemorrhages. **B** FFA shows upper temporal ischemic branch retinal vein occlusion (RVO) with peripheral phlebitis and telangiectasia. **C** SCP shows upper temporal non perfusion areas. **D** DCP shows diffuse ischemia. **E**, **F** 8 × 8 mm OCTA image of the SCP and DCP shows capillary changes, capillary network disorganization and upper temporal capillary non perfusion areas corresponding to branch RVO (arrows).

**Fig. 3:**
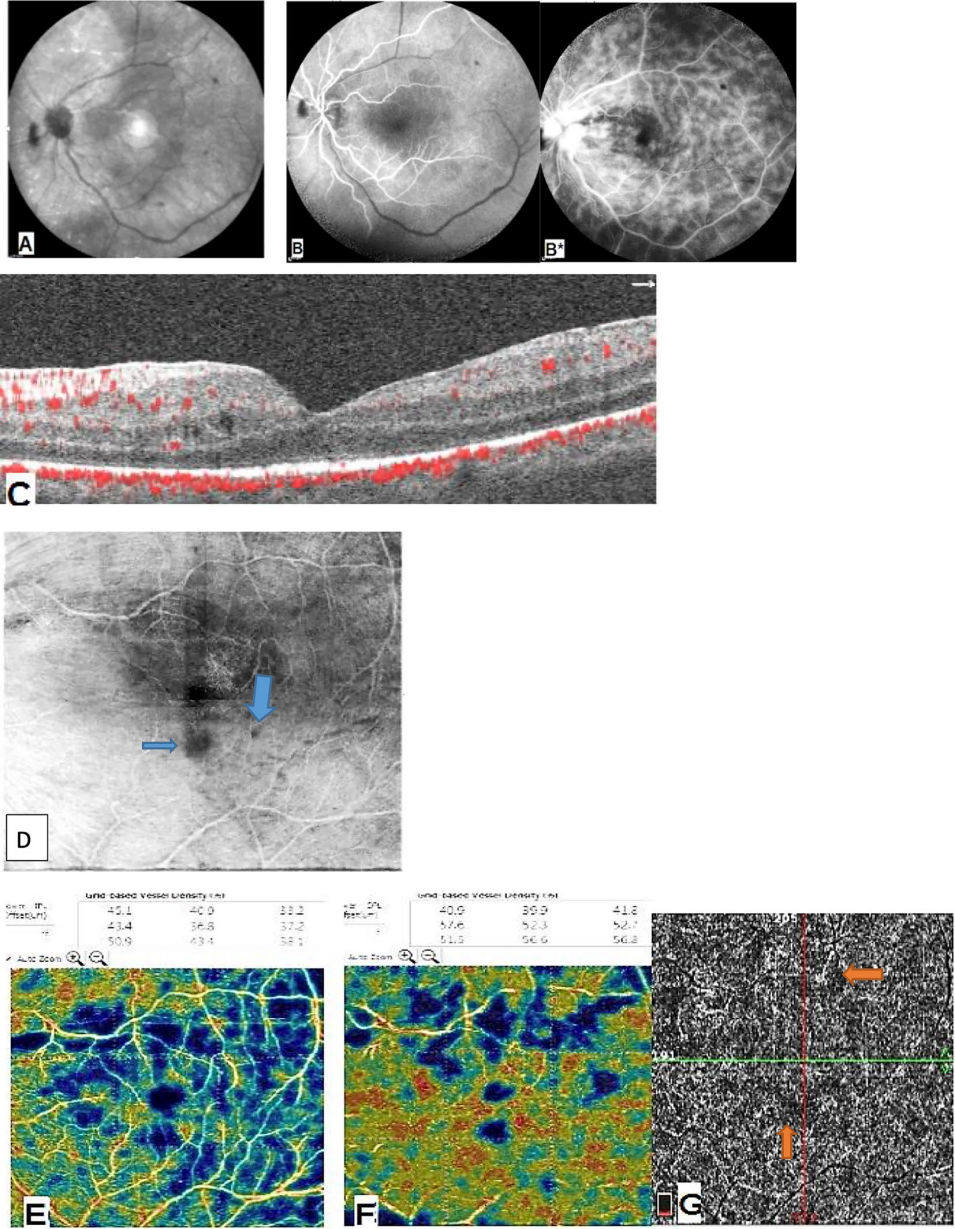
Patient with active sarcoidosis **A** Infra-red photo shows attenuated vessels. **B** FFA arterial phase. B* FFA late phase shows active vasculitis, optic disc leakage and macular edema. **C** Structural B-scan shows intra retinal cystoid spaces. **D **Enface image shows cystoid spaces (arrows) without evidence of back shadowing effect from vitreous floaters. **E**, **F** SCP and DCP respectively show ischemia. **G** Choriocapillaris layer shows areas of choroidal voids (arrows).

**Fig. 4:**
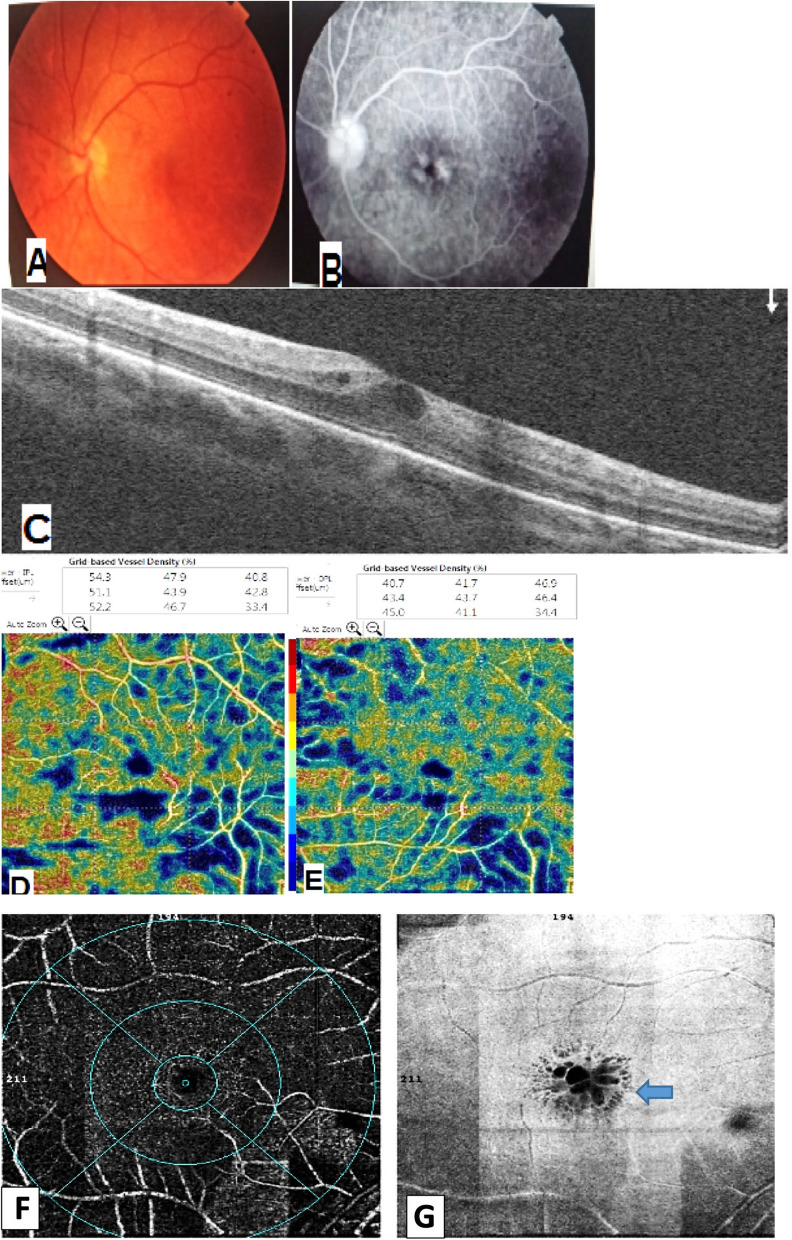
Patient with active sarcoidosis **A** Color photo shows lost foveal reflex **B** FFA shows hot optic disc, macular edema and peripheral vascular leakage. **C** Structural B-scan shows intra retinal cystoid spaces. **D**, **E** SCP and DCP respectively show ischemia more in the deep capillary plexus. **F** OCTA image of the DCP shows capillary changes and areas of capillary hypoperfusion **G** Enface image of the DCP shows well-defined black cystoid spaces

OCTA images showed areas of capillary non-perfusion / hypo perfusion in 30 eyes (100.0%), capillary changes in 15 eyes (50.0%), choroidal voids due to ischemia or infiltration in 11 eyes (36.7%), disorganization of the superficial and deep capillary network in 9 eyes (30.0%), and intraretinal cystoid spaces in 8 eyes (26.6%) (Figs. 1, 2, 3, 4).

### FFA findings in TB versus sarcoid patients

On comparing the FFA findings in both groups, statistical significance was only found when comparing the frequency of optic disc leakage, being higher in the sarcoidosis group (*P* value = 0.023) (Table 3) (Figs. 3, 4).

**Table 3:** FFA findings in TB versus sarcoid patients

	Tuberculosis		Sarcoidosis		*P* value
	Count	%	Count	%	
Macular edema	6	54.5	5	26.3	0.238
Optic disk leakage	1	9.1	10	52.6	0.023
Areas of active choroiditis	4	36.4	5	26.3	0.687
Macular ischemia	2	18.2	4	21.1	1.000
Active vasculitis	3	27.3	7	36.8	0.702
Inactive vasculitis	5	45.5	1	5.26	0.016
Peripheral ischemia	5	50.7	5	26.3	0.425

Vasculitis was found in 8 TB patients and 8 sarcoid patients, being occlusive and associated with peripheral ischemia in 5 patients in each group

### OCTA angiography findings in TB versus sarcoid patients

Areas of capillary non-perfusion/hypo perfusion were detected in all 11 eyes (100.0%) of the TB group and in all 19 eyes (100.0%) of the sarcoidosis group, regardless the state of the uveitic activity. Capillary changes (areas of rarefied capillaries) were detected in 6 eyes (54.5%) of the TB group and in 9 eyes (47.4%) of the sarcoidosis group. Choroidal voids due to ischemia or infiltration were detected in 5 eyes (45.5%) of the TB group and in 6 eyes (31.6%) of the sarcoidosis group. Intraretinal cystoid spaces were detected in 4 eyes (36.4%) of the TB group and in 4 eyes (21.1%) of the sarcoidosis group. The aforementioned comparisons were statistically insignificant.

On comparing quantitative parameters determined by OCTA, it was found that vascular densities in the DCP were significantly lower in the TB group in the mean whole image VD, mean inferior VD, mean nasal VD and mean temporal VD (Table 4).

**Table 4:** OCTA angiography quantitative changes in TB versus sarcoid patients

	Tuberculosis	Sarcoidosis	*P* value
FAZ (mm^2^) (mean)	0.28564	0.28564	0.444
SCP whole image VD (mean)	45.391	45.232	0.916
SCP foveal VD (mean)	42.745	39.384	0.072
SCP superior VD (mean)	46.027	46.611	0.759
SCP inferior VD (mean)	46.009	46.442	0.804
SCP nasal VD (mean)	47.964	48.011	0.981
SCP temporal (mean)	42.573	43.158	0.785
DCP whole image VD (mean)	40.200	44.763	0.024
DCP foveal VD (mean)	46.336	49.958	0.076
DCP superior VD (mean)	39.918	44.416	0.085
DCP inferior VD (mean)	39.682	44.726	0.020
DCP nasal VD (mean)	39.845	47.363	0.015
DCP temporal VD (mean)	45.091	50.300	0.016

### Comparing OCTA and FFA findings in all patients

FFA showed macular ischemia in only 6 eyes (20.0%) of all patients while OCTA showed areas of macular non-perfusion/hypo perfusion in all eyes (100%). It also showed macular edema in 11 eyes (36.7%), whereas OCTA showed intraretinal cystoid spaces in only 8 eyes (26.7%) of all patients. These findings were statistically significant (*P* =  < 0.001).

On attempting to associate the presence of parafoveal and perifoveal (the area 1–6 mm from the center of the fovea) ischemia on OCTA in the SCP and DCP with the disease type, treatment, activity and FFA findings, no statistically significant associations were found in either group.

### Correlating VD and BCVA

There was a positive but insignificant correlation between the whole image vascular density in the SCP and the mean BCVA of all patients (*R*- value = 0.359 and the *P*-value = 0.052) and between the whole image vascular density in the DCP and the BCVA (*R* = 0.143, *P* = 0.451).

## Discussion

In this work we analyzed the microvascular qualitative and quantitative changes detected by OCTA in 30 eyes with sarcoid or tuberculous-associated posterior uveitis.

This study showed that, by OCTA, multiple changes could be detected: all eyes had areas of capillary non-perfusion/hypo perfusion (even if activity was not clinically or angiographically detected), while 50% also had capillary changes in the form of areas of rarefied/telangiectatic capillaries. Likewise, Kim et al*.* [12] demonstrated that in contrast with healthy controls, uveitis subjects had distinct areas of qualitatively impaired retinal perfusion in the non-segmented retinal layer (NS-RL), both in the absence and presence of macular edema [12].

In our study we observed changes in the choriocapillaris layer, in 45.5% of the TB group and 31.6% eyes of the sarcoidosis group, in the form of ‘flow-voids’ which may be due to ischemia or infiltration (Figs. 1, 3, 4). This was explained by Cerquaglia et al. [13], as active granulomata, chronic tissue damage secondary to previously active granulomata (mechanically), or the presence of focal choroidal arteriolitis [13].

Agarwal et al. [14] also reported an increase in the areas of ‘flow-void’ by OCTA, which corresponded to active infiltrates found in 5 patients with tuberculous serpiginous like choroiditis, who had developed paradoxical worsening upon initiation of anti-tuberculous therapy. Thus, they concluded that OCTA may provide a simple, fast, non-invasive and high-resolution alternate imaging method to document progressive or recurrent choriocapillaris hypoperfusion, which is essential in the monitoring and follow up of eyes with choroiditis [14].

When documenting areas of flow voids, we excluded artifacts or loss of transmission by correlating them with their corresponding structural en face images and the cross-sectional OCT scans as recommended by Mahendradas et al. [15]

When comparing the VD in our patients (in the SCP and DCP), it was less than the normative data determined by Coscas et al. [11]. Thus, our study showed macular hypoperfusion in all patients, being even more evident in the DCP (Figs. 1, 2, 3, 4).

Likewise, Emre et al. compared 32 eyes of Behçet uveitis (during the inactive period of the disease as determined by conventional imaging techniques i.e. FFA) and 30 eyes of healthy controls. OCTA revealed microvascular changes such as parafoveal capillary telangiectasia and capillary retinal hypoperfusion despite the absence of activity and showed that the capillary vessel density of the Behçet group was significantly lower than in the control group. In addition, the DCP was affected more than the SCP in these patients***.*** Thus, the authors concluded that OCTA was more reliable than FA in monitoring patients and detecting early risk of central loss of vision [16].

The deep plexus has been shown in prior OCTA-based studies to be more vulnerable to impaired blood flow. This was implicated to be the result of its location in the “water shed region” at the termination of retinal capillary units [13, 13].

On comparing the qualitative and quantitative changes detected by OCTA in the TB group and the sarcoidosis group, our study showed that there was no statistically significant difference between the findings except in the vascular density of the DCP in the whole enface image and in the inferior, nasal and temporal sectors; being lower in the TB group.

Both diseases share many common features as they are both granulomatous and both can cause periphlebitis but in sarcoidosis occlusive periphlebitis was less common than in TB (Fig. 1). Tuberculous retinal periphlebitis is typically an obliterative periphlebitis and tends to cause hemorrhagic infarction of the retina [18]. This may explain the evident significant DCP ischemia detected by OCTA in the TB group and may help in differentiation between the two entities, which often pose a diagnostic dilemma as they share many clinical findings and their investigations (laboratory or radiological) are not always conclusive.

The comparison between the qualitative findings found on OCTA and FFA in this study, revealed that all eyes (100%) showed areas of para and perifoveal non-perfusion/hypo perfusion by OCTA. However, on FFA, only 6 eyes (20.0% of all patients) showed macular ischemia (highly significant on comparison). Thus, OCTA may be superior to FFA in detection of macular ischemia and also can quantify the ischemia in numerical values (capillary VD %) and localize the abnormalities by segmentation of the retinal and choroidal layers. These advantages are lacking in FFA devices.

In an OCTA study on patients with placoid pattern of TB, areas of choriocapillaris flow deficit detected on OCTA were correlated anatomically with ischemic lesions on FFA and ICGA, but were more extensive [19]. This was similar to our study and correlating with ICGA findings, although useful in these two entities due to their choroidal affection, is unfortunately not available in our region.

In 2017, Khairallah et al. described OCTA findings in eyes with active Behçet’s uveitis. They determined, as we did in our study, that OCTA allowed better visualization and characterization of parafoveal microvascular changes than FA, such as disruption of the capillary arcade, areas of retinal non-perfusion, and capillary abnormalities [20].

On the other hand, in our study FFA proved to be superior in detection of macular edema. FFA images revealed macular edema (leakage from an unhealthy capillary bed) was in 11 eyes (36.7%), whereas OCTA showed intraretinal cystoid spaces (confirmed by sequential en face images and corresponding SD OCT) in only 8 eyes (26.7%) of all patients (*P* =  < 0.001).

FFA remains indispensable in diagnosis and monitoring peripheral vasculitis, as seen in 8 of our TB patients and 8 of the sarcoid patients. Abucham-Neto et al., evaluated nineteen eyes with retinal vasculitis (2 eyes were associated with sarcoidosis) and reported that OCTA is unable to detect clear signs of active inflammation around the affected vessels like signs of vascular sheathing and perivascular leakage on FFA [21].

Our study showed that correlating parafoveal and perifoveal ischemia (detected by OCTA) in the SCP and DCP with the disease type, treatment, and activity was statistically insignificant. This may be related to the chronic and slowly progressive course of the ocular granulomatous inflammation in both groups. This may explain why ischemia is found in the TB and sarcoid eyes regardless whether they are treated or not and whether active or inactive [22].

The correlation between retinal vessel density and visual acuity remain unclear. Prior studies have reported significant negative correlations, while others did not find any correlation between both [17].

Our study found that there is positive, but statistically insignificant correlation between the vascular density in the SCP, DCP and the BCVA. This was contrary to our expectation that the vision would have been significantly correlated to the VD in DCP, as it was more affected than the SCP in our patients. This may be explained by our finding that the hypoperfusion found in the DCP may not necessarily manifest in ischemic damage of the outer retinal layers, as shown in Fig. 3.

Limitations of our study include the lack of differentiation of active/inactive and treated/untreated patients when comparing the FFA, OCTA qualitative and quantitative findings. Likewise, comparing the changes in these findings, following treatment of the active patients, would help further investigate the predictive value of OCTA in posterior uveitis and possibly allow a more significant correlation with the functional outcome (BCVA).

## Conclusion

This study is one of the few studies comparing OCTA findings in two of the most common causes of granulomatous posterior uveitis.

This study confirms the role of OCT angiography in detection of qualitative and quantitative microvascular changes in tuberculous- and sarcoid-associated uveitis and shows that OCTA is superior to FFA in detection of macular ischemia. On the other hand, FFA remains an essential complementary tool to detect activity of posterior uveitis and peripheral retinal vasculitis.

Although FFA is indispensable in demonstrating inflammatory activity in the posterior segment, OCTA can be used in follow up of patients and may be the only choice when FFA is contraindicated or refused. The presence of ischemia and lower vascular density found in the TB group may be a factor aiding in the differentiation between the two often overlapping entities.

## Data Availability

Data have not been placed in a repository but it is available upon request.
